# Case–case–control study of risk factors for carbapenem-resistant Enterobacterales infections among hospitalized patients

**DOI:** 10.1017/ash.2022.244

**Published:** 2022-07-14

**Authors:** David M. Stuever, Amy K. Ferketich, Jiyoung Lee, Kurt B. Stevenson, Thomas E. Wittum

**Affiliations:** 1 Division of Epidemiology, College of Public Health, The Ohio State University, Columbus, Ohio; 2 Division of Environmental Health Sciences, College of Public Health, The Ohio State University, Columbus, Ohio; 3 Division of Infectious Diseases, Department of Internal Medicine, The Ohio State University Wexner Medical Center, Columbus, Ohio; 4 Department of Veterinary Preventive Medicine, The Ohio State University, Columbus, Ohio; 5 Infectious Diseases Institute, The Ohio State University, Columbus, Ohio

## Abstract

**Objective:**

To identify important risk factors for carbapenem-resistant Enterobacterales (CRE) infections among hospitalized patients.

**Design:**

We utilized a case–case–control design that compared patients with CRE infections to patients with carbapenem-susceptible Enterobacterales (CSE) infections and randomly selected controls during the period from January 2011 through December 2016.

**Setting:**

The study population was selected from patients at a large metropolitan tertiary-care and instructional medical center.

**Patients:**

Cases of CRE were defined as initial admission of adults diagnosed with a bacterial infection of an Enterobacterales species resistant clinically or through sensitivity testing to carbapenems 48 hours or more after admission. Cases of CSE were selected from the same patient population as the CRE cases within a 30-day window for admission, with diagnostic pathogens identified as susceptible to carbapenems. Controls were defined as adult patients admitted to any service within a 30-day window from a CRE case for >48 hours who did not meet either of the above case definitions during that admission.

**Results:**

Antibiotic exposure within 90 days prior to admission and length of hospital stay were both associated with increased odds of CRE and CSE infections compared to controls. Patients with CRE infections had >18 times greater odds of prior antibiotic exposure compared to patients with CSE infections.

**Conclusions:**

Antibiotic exposure and increased length of hospital stay may result in increased patient risk of developing an infection resistant to carbapenems and other β-lactams.

In 2017, carbapenem-resistant Enterobacterales (CRE) caused an estimated 13,100 infections and 1,100 deaths annually in the United States, with case-fatality rates nearing 50% in some patient populations.^1^ CRE infections are generally resistant to multiple clinically relevant classes of antibiotics, including β-lactams and have been classified by the US Centers for Disease Control and Prevention (CDC) as an urgent public health threat.^1,2^

Bacterial resistance to β-lactams and carbapenems is frequently mediated by β-lactamase genes located on mobile genetic elements that can be transferred horizontally.^3,4^ Although multiple carbapenemase genes have been reported in the United States, *bla*_KPC_ has emerged and spread rapidly both domestically and worldwide.^3,5^ Within healthcare and long-term care facilities, organisms expressing resistance to carbapenems are becoming increasingly more common and have been recovered from humans,^6,7^ animals,^8–11^ and the environment.^12,13^

Risk factors associated with contracting carbapenem-resistant *Klebsiella* spp have been reported.^14–16^ *Klebsiella* spp have been the most prominent host for *bla*_KPC_, but recently other enteric organisms, including *Enterobacter* spp, have emerged as clinically important CRE.^6^ For this study, we compared patients diagnosed with CRE infections to patients diagnosed with carbapenem-susceptible infections and control patients to further characterize important risk factors for CRE infections.

## Methods

### Study design

In this study, we utilized a case–case–control design^17^ to identify risk factors for adult patients diagnosed with CRE infections admitted for >48 hours to a large, metropolitan, instructional medical center between January 2011 and December 2016. Cases of CRE infection were compared to patients diagnosed with carbapenem-susceptible Enterobacterales (CSE) infections and to controls randomly selected from among all patients admitted within 30 days of each CRE case. Patient records selected for this study were deidentified prior to analysis.^15,17–19^

### Case definitions

Cases of CRE were defined as initial admission of adults admitted between January 2011 and December 2016 who were diagnosed with a bacterial infection of an Enterobacterales species resistant clinically or through sensitivity testing to carbapenems ≥48 hours after admission.

Cases of CRE were identified through a database maintained by the medical center’s antimicrobial stewardship program to track patients having an antibiotic-resistant infection. Generally, CRE infections are identified phenotypically based on carbapenemase production,^20–23^ or confirmed to harbor a known carbapenemase gene.^24^

Cases of CSE were selected from the same patient population as the CRE cases within a 30-day window for admission, with diagnostic pathogens identified as susceptible to carbapenems. Eligible CSE cases were independently identified by the medical center data warehouse and a subset was randomly selected for inclusion in the study.

Controls were defined as adult patients admitted to any service within a 30-day window from a CRE case for >48 hours who did not meet either of the above case definitions during that admission. All matched controls were randomly selected from the population of eligible patients by the information warehouse.

This project was determined to be exempt by The Ohio State University Institutional Review Board (study no. 2016E0677) and was granted a full waiver of HIPAA authorization by the HIPPA Privacy Board.

### Data collection, management, and analysis

The following information was collected from the medical records of patients from all 3 patient groups: date of admission, age at admission, onset date of infection, sex, length of hospital stay (LOS), race, ethnicity, ZIP code, self-identified history of tobacco and alcohol use, Charlson score at admission, use of dialysis and catheter during the admission, prescription for antibiotics in the 90 days prior to admission, and organism with which the patient was infected.

The target sample size for this study was 110 per study group using α = 0.05 and β = 0.20 based on an expected prevalence of exposure to β-lactam antibiotics of 15% in controls.

Documented use of antibiotics for the previous 90 days prior to the date of admission was recoded as yes or no and the general class of antibiotics (β-lactams, others). The total number of antibiotics prescribed in the previous 90 days for any individual was recorded both by antibiotic class and total number.

The self-reported use of either alcohol or tobacco products on or nearest the date of admission was used to classify patients as alcohol drinkers (yes or no) or smokers (yes or no). Any positive response to use was recoded as a yes for analysis. The use of dialysis and catheters during admission or in the previous 90 days as well as any surgeries recorded in the medical record to have occurred within the previous 90 days were recorded.

The Charlson score was coded as a numeric score for analysis.^25^ The LOS was calculated as the number of days from admission to discharge. Age was recoded into 3 groups for analysis: 18–44 years, 45–64 years, and >65 years.^15^ The ZIP code of the patient’s home address was recategorized into urban or rural using the 2013 Rural–Urban Continuum Codes from the US Department of Agriculture Economic Research Service.^26^

Data management and analysis were completed using Stata version 13.1 software (StatCorp, College Station, TX). Simple and multiple logistic regression models were based on previously identified risk factors related to CRE infection.^15^ Univariable logistic regression models were analyzed to determine the unadjusted influence of independent variables on the outcome. Any clinically valid potential risk factors were reviewed independently and included in the full model if found to be plausible and statistically significant in the simple models. A full multivariable explanatory model was created using α = 0.10 as the initial inclusion criteria and α = 0.05 cutoff value for the final full model.

Backward stepwise multivariable logistic regression models were fit to develop the final model. For the multivariable model, an initial bivariable model was created with previous β-lactam use forced into the model as the primary factor of interest in this study and then adding all significant factors with an initial α < 0.10. A second model was then created using the significant variables from the initial model, retaining the previous use of β-lactams, and with entry and removal criteria lowered to 0.05 and 0.10, respectively. Finally, a model was developed that removed β-lactams specifically and added prior prescriptions for any class of antibiotic in the previous 90 days, using the same criteria for entry and removal of variables.

## Results

In total, 432 CRE occurrences were identified over the 6-year study period in the initial medical-center data set, retaining only initial occurrences resulted in 81 unique CRE cases for analysis. To these cases were matched 87 CSE cases and 89 controls. Descriptive statistics for the study groups are summarized in Table 1. Differences among study groups were identified for proportion males (χ^2^ = 7.90, *P* =.019), age at admission (F = 1.75; *P* = .002), and LOS (F = 4.58; *P* < .0001) (Table 1). A summary of initial logistic models comparing each of the case types to controls is shown for CRE in Table 2 and for CSE in Table 3.

**Table 1.** Demographic Information for 81 Patients With Carbapenem-Resistant Enterobacterales (CRE) Infections, 87 Patients With Carbapenem-Susceptible Enterobacterales (CSE) Infections, and 89 Control Patients at a Large, Metropolitan, Tertiary-Care Hospital

Characterstic	CRE (n=81)	CSE (n=87)	Control (n=89)
Sex, male, no. (%)	52 (64.2)	45 (51.7)	38 (42.7)
**Race, no. (%)**			
White	55 (67.9)	65 (74.7)	67 (75.3)
Black	21 (25.9)	17 (19.5)	14 (15.7)
Other	4 (4.9)	5 (5.8)	7 (7.9)
Not Hispanic/Latino	80 (98.8)	86 (98.9)	87 (97.8)
Charlson score, mean (SD)	3.25 (3.2)	3.46 (3.1)	2.54 (3.5)
**Age group, no. (%)**			
18–44 y	22 (29.0)	17 (22.4)	37 (48.7)
45–64 y	32 (33.3)	41 (42.7)	23 (24.0)
≥65 y	27 (31.8)	29 (34.1)	29 (34.1)
Length of hospital stay, mean d (SD)	33.4 (32.1)	23.3 (43.1)	6.9 (8.7)
Prior antibiotics, mean no. (SD)	5.0 (2.7)	3.8 (2.0)	3.1 (2.3)
Rural home, no. (%)	12 (15.8)	13 (16.1)	21 (27.3)

**Table 2.** Results of Ordinary Logistic Regression Models for Estimating the Unadjusted Odds Ratios for Various Demographic and Clinical Variables for 81 Patients With Carbapenem-Resistant Enterobacterales (CRE) Infections Compared to 89 Control Patients

Risk Factor	Odds Ratio	95% Confidence Interval	Logistic Regression χ^2^	*P* > χ^2^	No.
Sex, male	2.41	1.30–4.47	7.94	.0048	170
Smoker	1.83	0.99–3.37	3.80	.0512	170
**Age (<45 y)**	Ref	…	5.03	.0809	170
45–64 y	2.34	1.10–4.96			
>64 y	1.57	0.74–3.29	…	…	…
**Race (white)**	Ref	…	2.64	.2676	169
Black	1.83	0.85–3.92	…	…	…
Other	0.87	0.26–2.89	…	…	…
Surgery	42.6	17.3–105	99.28	<.0001	165
Ventilator	4.88	2.31–10.3	19.28	<.0001	170
Antibiotic use, any class	51.1	14.9–176	83.65	<.0001	170
Drink alcohol	0.94	0.73–1.21	0.21	.6469	161
Rural home	0.5	0.23–1.11	3.01	.0826	153
Length of stay, per day	1.19	1.13–1.26	105.51	<.0001	170
Charlson score at admission	1.07	0.97–1.18	1.82	.1774	159
Charlson ≥ 2	1.83	0.97–3.47	8.69	.0032	159
Prescribed β-lactam	12.6	6.05–26.3	55.96	<.0001	170
Antibiotics prescribed, no.	1.36	1.12–1.66	11.38	.0007	111

**Table 3.** Results of Ordinary Logistic Regression Models for Estimating the Unadjusted Odds Ratios (OR) for Various Demographic and Clinical Variables for 87 Patients With Carbapenem-Susceptible Enterobacterales (CSE) Infections Compared to 89 Control Patients

Risk Factor	Odds Ratio	95% Confidence Interval	Logistic Regression χ^2^	*P* > χ^2^	No.
Sex, male	1.44	0.79–2.61	1.44	.2300	176
Smoker	0.94	0.51–1.73	0.04	.8457	176
**Age (<45 y)**	Ref	…	12.70	.0018	176
45–64 y	3.88	1.80–8.37	…	…	…
>64 y	2.18	1.01–4.71	…	…	…
**Race (white)**	Ref	…	0.65	.7224	175
Black	1.25	0.57–2.75	…	…	…
Other	0.74	0.22–2.44	…	…	…
Surgery	0.63	0.22–1.78	0.78	.2232	147
Ventilator	1.91	0.87–4.21	2.69	.1007	176
Antibiotic use, any class	2.79	1.51–5.14	11.11	.0009	176
Drink alcohol	0.94	0.73–1.21	0.23	.6291	165
Rural home	0.51	0.23–1.11	2.96	.0852	158
Length of stay, per day	1.09	1.06–1.14	39.42	<.0001	176
Charlson score at admission	1.09	0.99–1.20	3.06	.0804	157
Charlson ≥ 2	2.25	1.18–4.27	12.07	.0005	157
Prescribed β-lactam	2.44	1.29–4.61	7.83	.0051	157
Antibiotic prescribed, no.	1.17	0.94–1.47	2.00	.1577	81

### CRE compared to control patients

Risk factors that increased the unadjusted odds of CRE compared to controls were male sex (OR, 2.4; 95% CI, 1.3–4.47), age 45–64 years (OR, 2.3; 95% CI, 1.1–4.96), surgery (OR, 42.6; 95% CI, 17.3–105), use of a ventilator (OR, 4.9; 95% CI, 2.31–10.3), prescribed any antibiotic in the previous 90 days (OR, 51.1; 95% CI, 14.9–176), prescribed a β-lactam in the previous 90 days (OR, 12.6; 95% CI, 6.06–26.3). LOS (OR, 1.2; 95% CI, 1.13–1.26) and total number of antibiotic prescriptions in the 90 days prior to admission or diagnosis (OR, 1.36; 95% CI, 1.12–1.66) were also greater for CRE patients compared to controls (Table 2). The odds of a Charlson score at admission of ≥2 was not different between CRE and control patients, but it did meet the criteria for initial inclusion in developing a multivariable model.

### CSE compared to control patients

CSE patients were more likely than controls to be aged 45–64 years (OR, 3.9; 95% CI, 1.8–8.37) or >64 years (OR, 2.2; 95% CI, 1.01–4.71), to have been prescribed any class of antibiotics in the previous 90 days (OR, 2.8; 95% CI, 1.51–5.14), and to have been prescribed a β-lactam in the previous 90 days (OR, 2.4; 95% CI, 1.29–4.61). LOS was also longer for CSE patients than for control patients (OR, 1.09 per day; 95% CI, 1.06–1.14) (Table 3).

CSE patients were at greater odds of having a Charlson score ≥ 2 compared to control patients (OR, 2.25; 95% CI, 1.18–4.27). The use of a ventilator and having a rural residence also met the inclusion criteria for the final multivariable model.

### CRE compared to CSE patients

The unadjusted risk factors for CRE patients compared to CSE patients were being a current smoker, surgery, ventilator use, prescribed antibiotics, an increasing number of antibiotics prescribed, and a prescribed β-lactam (Table 4).

**Table 4.** Results of Ordinary Logistic Regression Models for Estimating the Unadjusted Odds Ratios (OR) for Various Demographic and Clinical Variables for 81 Patients With Carbapenem-Resistant Enterobacterales (CRE) Infections Compared to 87 Patients With Carbapenem-Susceptible Enterobacterales (CSE) Infections

Risk Factor	Odds Ratio	95% Confidence Interval	Logistic Regression χ^2^	*P* > χ^2^	No.
Sex, male	1.67	0.90–3.11	2.69	.1013	168
Smoker	1.94	1.05–3.60	4.53	.0333	168
**Age (<45)**	Ref	…	1.61	.4466	168
45–64 y	0.61	0.28–1.32	…	…	…
>64 y	0.72	0.32–1.64	…	…	…
**Race (white)**	Ref	…	1.04	.5940	168
Black	1.46	0.70–3.03	…	…	…
Other	1.18	0.32–4.29	…	…	…
Surgery	67.5	23.1–196	98.76	<.0001	144
Ventilator	2.55	1.31–4.96	7.85	.0051	168
Antibiotic use, any class	18.4	5.37–62.8	38.39	<.0001	168
Drink alcohol	1	0.75–1.34	0.00	.9865	160
Rural home	0.98	0.42–2.31	1.71	.1906	168
Length of stay, per day	1.01	0.99–1.02	3.45	.6320	168
Charlson score at admission	0.98	0.89–1.08	0.18	.6714	158
Charlson ≥ 2	0.83	0.43–1.60	0.31	.5779	158
Prescribed β-lactam	5.17	2.56–10.4	23.57	<.0001	168
Antibiotic prescribed, no.	1.22	1.04–1.42	7.02	.0080	132

CRE patients were at 18 times greater odds of previous antibiotic use of any class compared to CSE patients (OR, 18.35; 95% CI, 5.37–62.8); however, we detected a difference in the proportion of CRE and CSE patients who were prescribed antibiotics in the previous 90 days (*P* < .001). Additionally, CRE patients were at 5 times increased odds of use of a β-lactam compared to CSE patients (OR, 5.16; 95% CI, 2.56–10.4). Again, the proportion of CRE patients and CSE patients differed in the recorded use of β-lactams in the previous 90 days (*P* < .001).

### Multivariable models

With the previous use of β-lactams retained in the model, the only risk factor for both CRE and CSE patients compared to controls in the initial multivariable model was LOS. Age group was associated with CSE cases compared to controls, and surgery was associated with CRE cases compared to controls.

A second model with the criteria listed above removed age from the model, although age is not a modifiable risk factor. In this model prescription of a β-lactam was associated with a 5.4 times increase in odds (95% CI, 1.95–15.1) for CRE infection and a 2.7 times increase in odds (95% CI, 1.18–5.95) for CSE infection compared to controls. For both CRE and CSE patients, each additional day of admission was associated with an increased odds of infection of about 13%, whereas surgery increased odds of CRE infection by 12.6 times compared to controls (95% CI, 4.32–36.44) but was associated with a lower odds for CSE infection (95% CI, 0.07–0.74).

The final model was used to determine whether any prescribed antibiotics was associated with an increased odds of either CRE or CSE infection. Increasing prescriptions of antibiotics was significant for both CRE patients (OR, 1.65; 95% CI, 1.32–2.07) and CSE patients (OR, 1.34; 95% CI, 1.13–1.60). LOS was associated with an increased odds for both CRE patients (OR, 1.09; 95% CI, 1.05–1.14) and CSE patients (OR, 1.09; 95% CI, 1.04–1.13). Surgery (OR, 19.3; 95% CI, 5.91– 63.0) and a rural residence (OR, 0.23; 95% CI, 0.06–0.94) were only associated with CRE infection.

A model comparing CRE to CSE patients was built using the criteria listed above for the other multinomial models, and several factors were used including smoking, surgery, use of ventilator, LOS, any prescribed antibiotics, and prescribed β-lactam. Although all of these factors were associated in simple unadjusted models, none were significant following adjustment resulting in no multiple logistic regression models being created. Table 5 summarizes the risk factors associated with both CRE and CSE infections compared to controls in adjusted models.

**Table 5.** Adjusted Odds Ratios (ORs) From Final Multivariable Logistic Regression Models Comparing 81 Carbapenem-Resistant Enterobacterales (CRE) and 87 Carbapenem-Susceptible Enterobacterales (CSE) Patients to 89 Control Patients

Risk Factor	OR	95% CI	*P* Value
**CRE patients vs control model**			
Prescribed β-lactam	5.43	1.95–15.1	.001
Length of hospital stay, d	1.14	1.08–1.19	<.001
Surgery	12.6	4.32–36.44	<.001
**CSE patients vs control model**			
Prescribed β-lactam	2.65	1.18–5.95	.018
Length of hospital stay, d	1.13	1.08–1.18	<.001
Surgery	0.23	0.07–0.74	.014

## Discussion

In this study, we sought to describe and compare risk factors for infections of CRE and CSE infections in a hospital environment. Although the risk factors for both groups are generally thought to be similar,^16^ it has been previously demonstrated that recent antibiotics or β-lactam use is a risk factor for CRE.^27,28^ In this study, CRE patients had an increase of >12 times the odds of having been prescribed a β-lactam antibiotic compared to control patients. This finding could be attributed to patients with CRE having more serious illnesses at admission requiring more intensive treatment or patients being more likely to be admitted due to having previous infections that were not captured. Although these odds were halved in a multiple logistic regression model, we detected a clear association of recent use of antibiotics in general and β-lactams specifically and being diagnosed with CRE. Previous studies have reported similar results with respect to the prior use of antibiotics. In Gallagher et al,^15^ carbapenem-resistant *K. pneumoniae* patients were 5.9 times more likely to have used antibiotics in their adjusted model. McLaughlin et al^27^ concluded that carbapenem use was associated with resistance in Enterobacterales. In another case–case–control study, Marchaim et al^28^ reported an association between antimicrobial exposure and CRE. When compared to controls, extended-spectrum β-lactamase (ESBL) producing Enterobacterales and non–ESBL-producing Enterobacterales, previous use of antimicrobials was consistently associated with CRE.^28^ Risk factors for CSE infection were similar in multivariable models to CRE infection, though older age group was significant for CSE infection and not for CRE infections.

An important risk factor for both CRE and CSE infection compared to controls determined from the multivariable models was LOS. Each day increased the odds of being diagnosed with either CSE or CRE by ∼10% when adjusting for the previous β-lactam use. Patients with an extended LOS could be at higher risk of infection due to the increased contact time with the environment, healthcare providers and other patients. These findings are consistent with previous studies’ findings.^15,29–31^ Alternatively, infections may have resulted in a longer LOS. Unfortunately, the data collected for this study did not allow us to determine which of those scenarios was more likely.

Surgery was also associated with CRE infections compared to controls, though there were only 6 recorded surgeries in the control patients. Surgery generally increases LOS increasing the potential for infection during the stay, and an open wound increases risk of infection of any type. In a small study in Brazil, CRE was shown to be associated with mediastinitus following cardiac surgery,^32^ and with infection following liver transplantation.^33^

In this study, we detected some differences in risk factors for CRE and CSE infections. First, patients with any previous antibiotic use were shown to have >18 times increased odds of being infected with CRE over CSE. Additionally, CRE patients had 5 times greater odds of β-lactam use over CSE patients. Neither the mean Charlson score nor the proportion of those CRE and CSE patients with Charlson scores ≥2 at the time of admission were different. However, the type of comorbidities was not measured in this study and could have led to a difference in how patients were prescribed antibiotics.

This study had several limitations. This was a retrospective analysis using patient information from a medical system that may have been incomplete. This may have resulted in misclassification in our data. In addition, the number of study subjects for which we were able to obtain data was fewer than our target sample size, which likely reduced our ability to detect associations.

Future studies of this type may consider conducting more thorough reviews of medical records to determine specific residence type prior to admission and whether patients are coming from common locations that have seen patients previously diagnosed with either CRE or CSE. Long-term and other group care facilities may have increased incidence of infection in general and of resistant organisms specifically and determining where patients are being admitted from can be important factors in determining how to care for these patients upon admission to a medical facility. Although independent studies on the prevalence of resistant bacteria in healthcare and other environments have been conducted,^34–37^ it may be worthwhile to determine the incidence of infection within a healthcare facility and the relationship to isolates cultured from within the environment of that specific location, and how those factors relate to each other in time. It is also important to determine how the LOS from the date of admission to date of diagnosis affects different infections, and whether a lack of diagnosis would decrease the overall LOS.
